# Supplementary material for: An enzymatic activation of formaldehyde for nucleotide methylation
Source: Nat Commun. 2021 Jul 27;12:4542. doi: 10.1038/s41467-021-24756-8 (PMC8316439; doi:10.1038/s41467-021-24756-8)
Supplement: Supplementary file 1 — Supplementary Information [file 41467_2021_24756_MOESM1_ESM.pdf]

Supplementary Information

An enzymatic activation of formaldehyde for nucleotide methylation

Charles Bou-Nader[1,6†], Frederick W. Stull[2†], Ludovic Pecqueur[1†], Philippe Simon[1], Vincent Guérineau[3], Antoine Royant[4,5], Marc Fontecave[1], Murielle Lombard[1], Bruce A. Palfey[2] and Djemel Hamdane[1*]

[1]Laboratoire de Chimie des Processus Biologiques, CNRS-UMR 8229, Collège De France, Université Pierre et Marie Curie, 11 place Marcelin Berthelot, 75231 Paris Cedex 05, France

[2]Programs in Chemical Biology and the Department of Biological Chemistry, University of Michigan Medical School, 1150 West Medical Center Drive, Ann Arbor, Michigan 48109, United States.

[3]Université Paris-Saclay, CNRS, Institut de Chimie des Substances Naturelles UPR 2301, 1 avenue de la Terrasse, 91198 Gif-sur-Yvette, France

[4]Univ. Grenoble Alpes, CEA, CNRS, Institut de Biologie Structurale (IBS), Grenoble, France

[5]European Synchrotron Radiation Facility, Grenoble, France

[6]Present address: Laboratory of Molecular Biology, National Institute of Diabetes and Digestive and Kidney Diseases, Bethesda, MD 20892. USA.

†These authors contributed equally.

*Correspondence: djemel.hamdane@college-de-france.fr (D.H.)

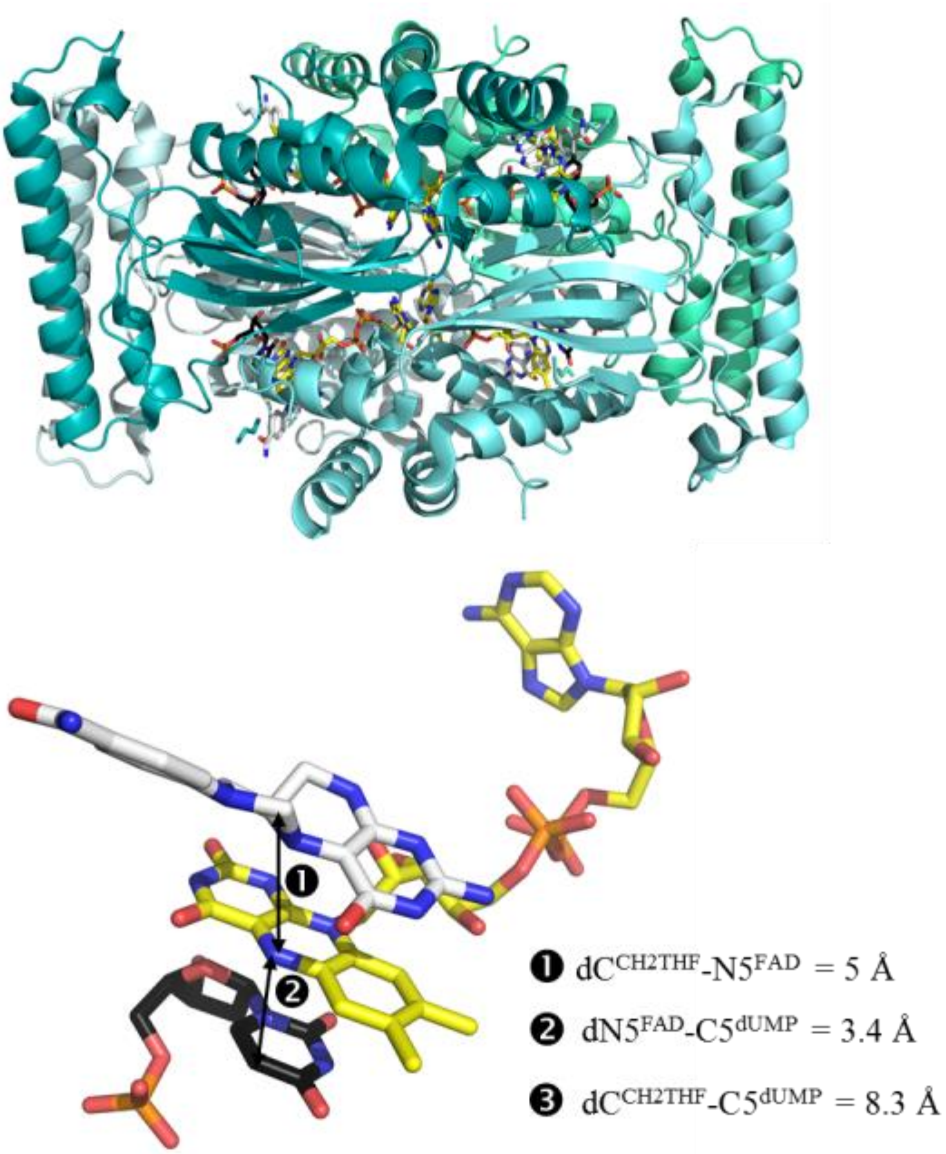

Supplementary Figure 1: Crystal structure of *T. maritima* ThyX in complex with dUMP and CH_2THF. Top: 3-D structure representation of homotetramer ThyX (PDB, 4gt9)[1]. The four equivalent active sites, each of them containing an oxidized FAD coenzyme (yellow sticks), a dUMP substrate (black sticks) and a CH_2THF cofactor (white sticks), are formed at the interface of three monomers. Bottom: Zoom on one of the four active sites showing how FAD, dUMP and CH_2THF are oriented in ThyX. The atomic distances between the reactive centers are indicated.

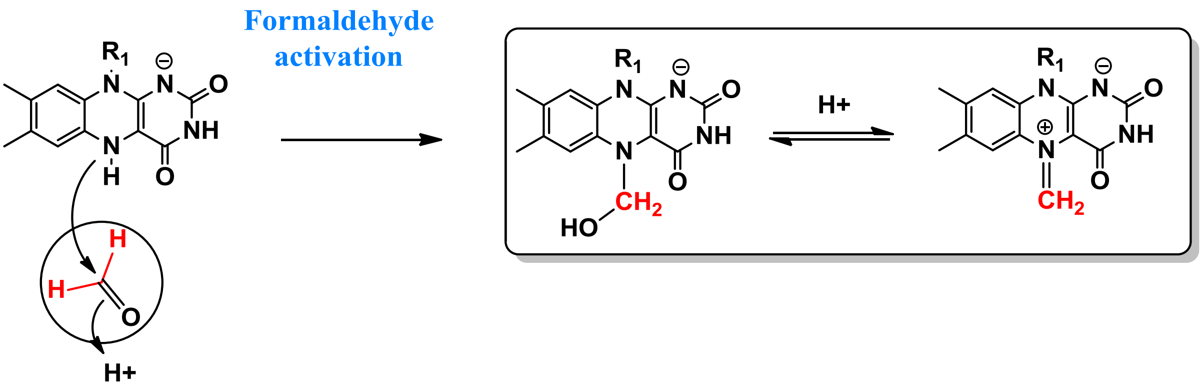

Supplementary Figure 2: Proposed mechanism for CH_2O activation by reduced FAD coenzyme. Nucleophilic N5 nitrogen of flavin hydroquinone, $FADH^-$, attacks CH_2O to generate a carbinolamine flavin species, which in theory should be in equilibrium with its iminium counterpart. So far, the latter is postulated as being the bona fide dUMP methylating agent[2].

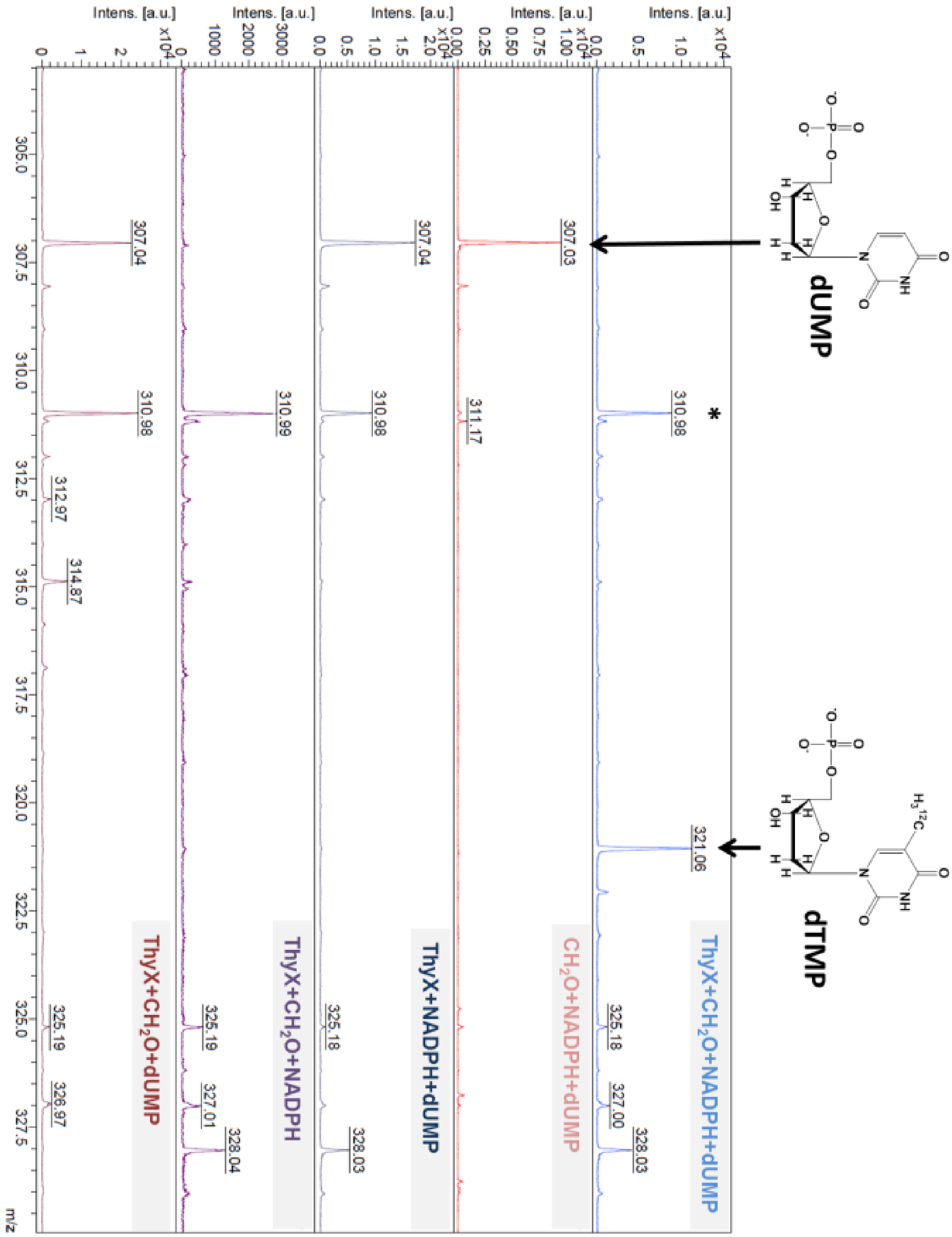
dUMP
dTMP
ThyX+CH2O+NADPH+dUMP
CH2O+NADPH+dUMP
ThyX+NADPH+dUMP
ThyX+CH2O+NADPH
ThyX+CH2O+dUMP
Intens. [a.u.]
m/z

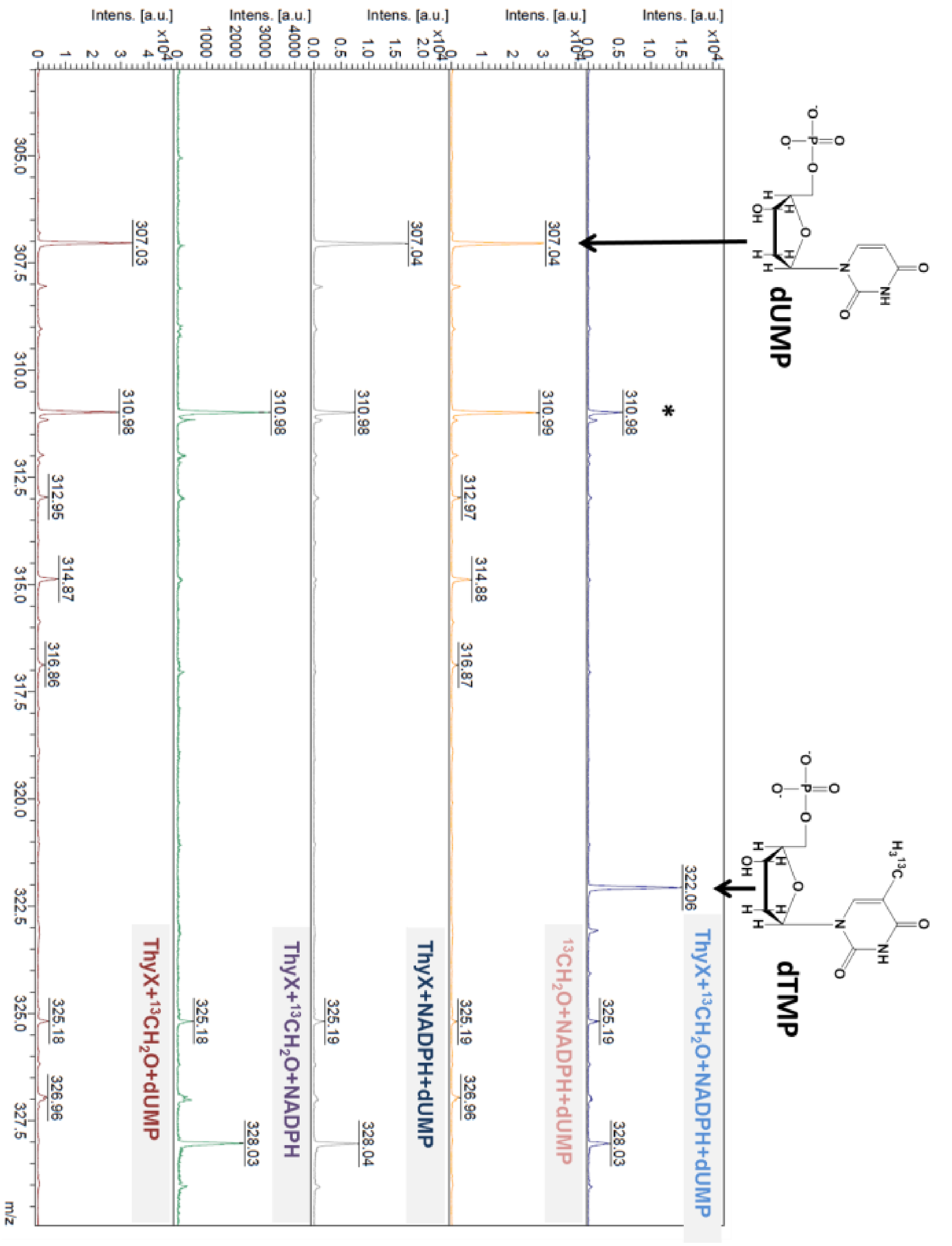

Supplementary Figure 3: MALDI-MS spectra of dTMP produced by ThyX in the presence of unlabeled and ^{13}C-labeled CH_2O. Formation of dTMP is observed exclusively in the presence of ThyX + CH_2O + NADPH + dUMP. Removal of one of these components from the activity test leads to the absence of dTMP formation. dUMP (307.04 *m/z*), [^{12}C7]-dTMP (321.06 m/z), [^{13}C7]-dTMP (322.06 *m/z*). (*) undetermined species from the matrix.

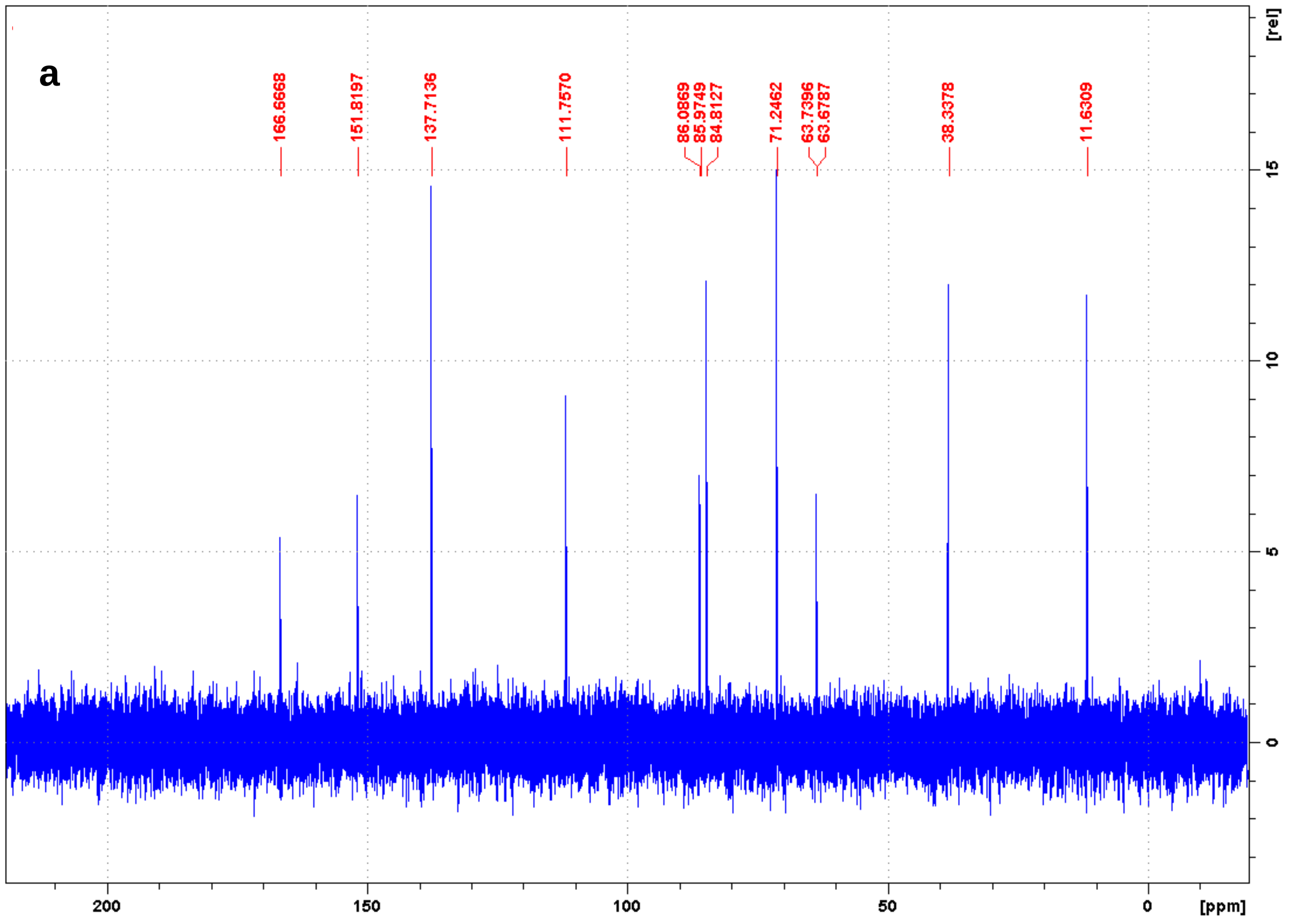
a
166.6668
151.8197
137.7136
111.7570
86.0869
85.9749
84.8127
71.2462
63.7396
63.6787
38.3378
11.6309
[rel]
15
10
5
0
200
150
100
50
0
[ppm]

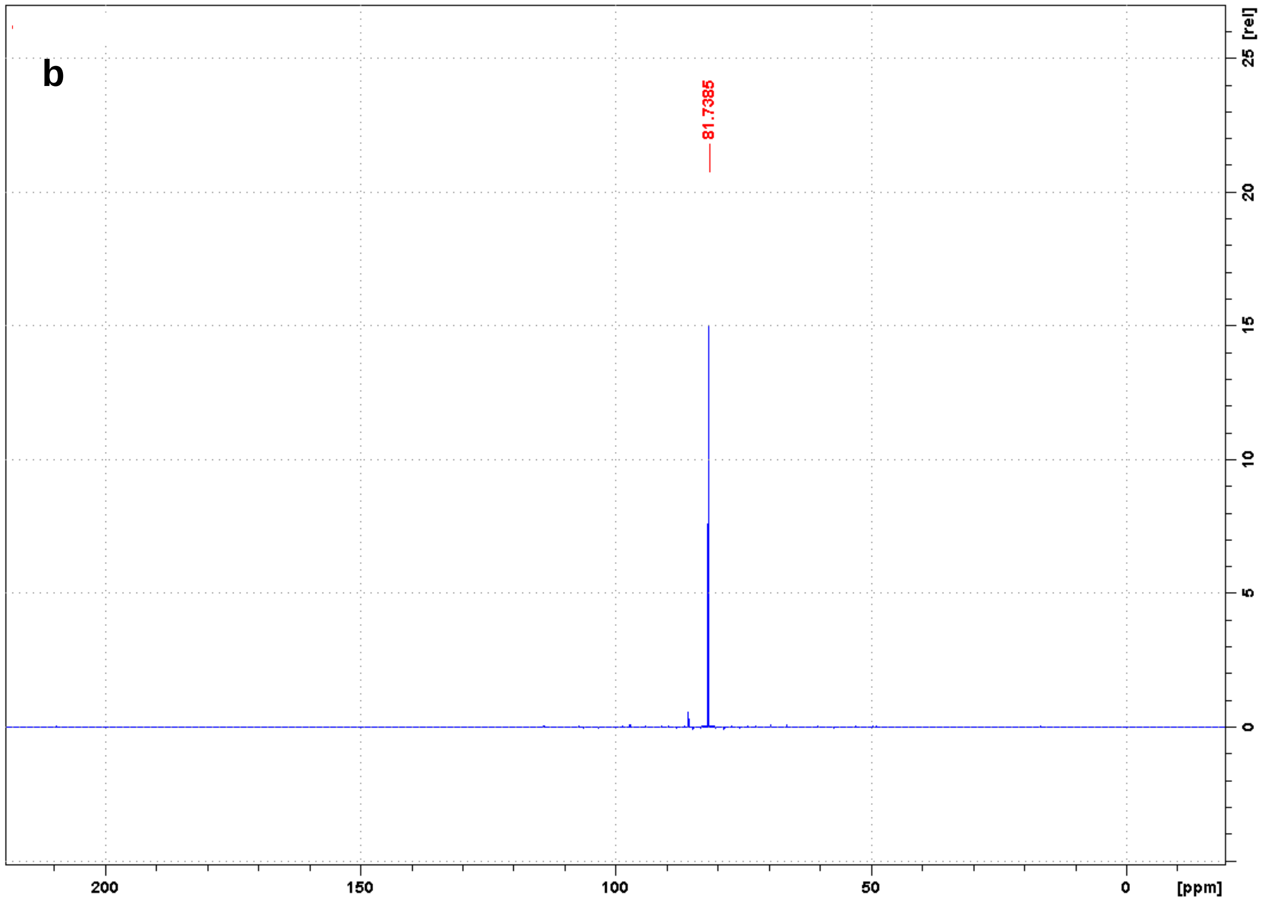
b
81.7385
[rel]
25
20
15
10
5
0
200
150
100
50
0
[ppm]

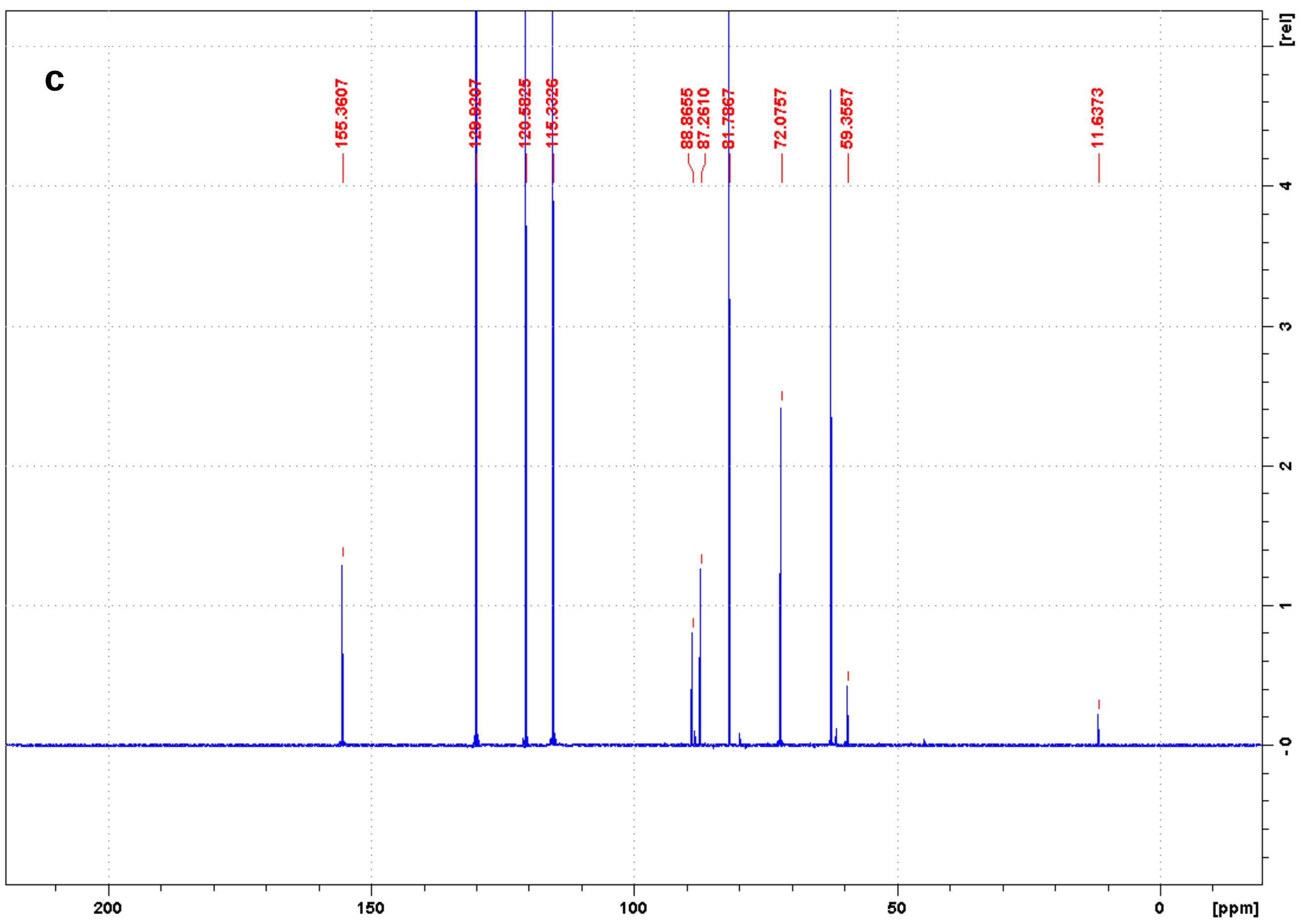
c
155.3607
129.9207
120.5925
115.3326
88.8655
87.2610
81.7867
72.0757
59.3557
11.6373
200
150
100
50
0
[ppm]
[rel]

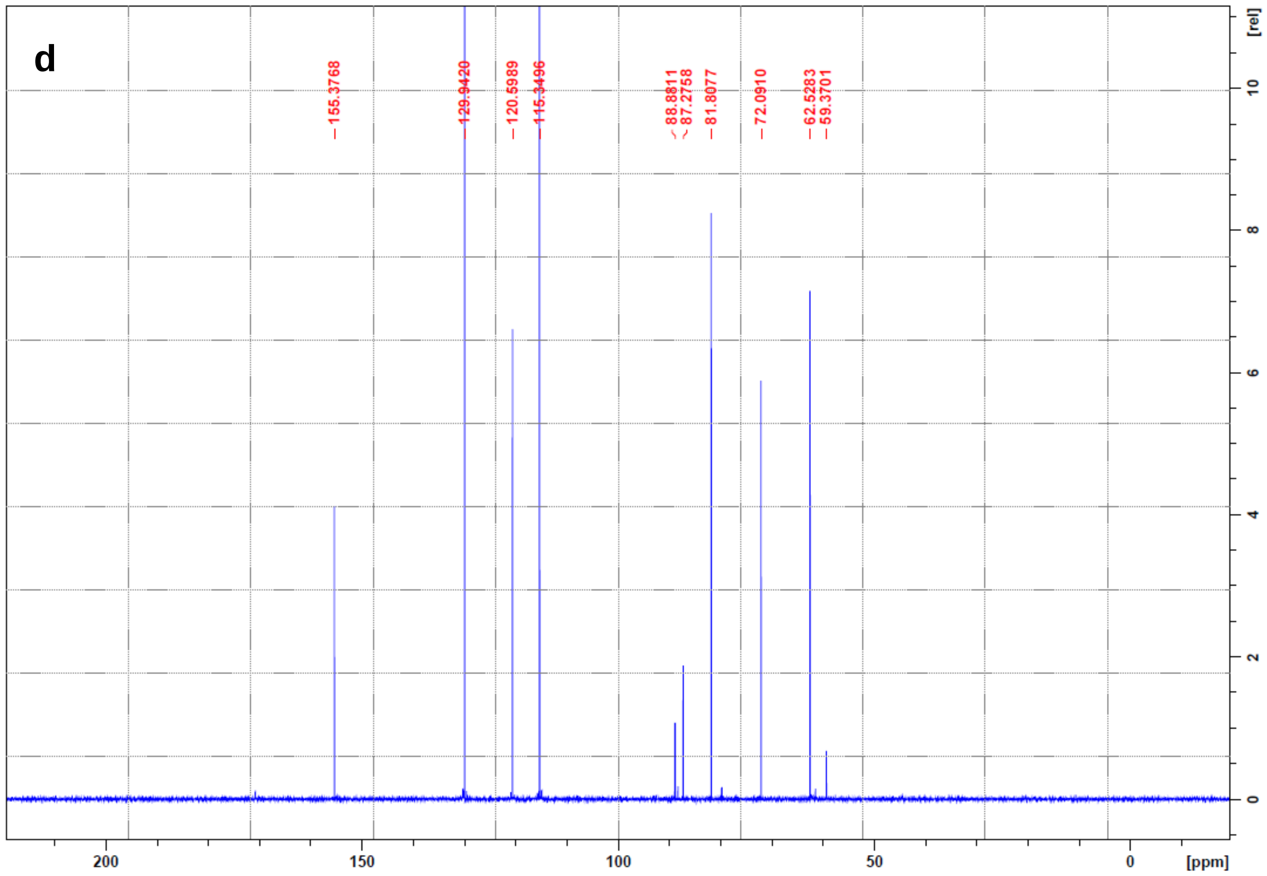
d
155.3768
129.9420
120.5989
115.3496
88.8811
87.2758
81.8077
72.0910
62.5283
59.3701
200
150
100
50
0
[ppm]
[rel]

Supplementary Figure 4: 1D NMR spectra to probe ThyX-dependent methylation of dUMP. 1D ^{13}C-NMR spectra of **(a)** dTMP**, (b)** ^{13}C-labelled CH_2O**, (c)** phenol-extracted products of ThyX activity test with ThyX or **(d)** without ThyX**.** Activity tests were performed in the presence of ThyX, NADPH, dUMP and CH_2O. The intense peaks, at 115, 121, 129 and 155 ppm arise from phenol in **c** and **d.** The peak at 81 ppm arise from formaldehyde in **b**, **c** and **d**.The peak at 62 ppm probably arise from Tris-Cl buffer in **c** and **d**. The other minor peaks at 59, 72, 87 and 88 ppm could not be attributed but are present in **c** and **d**, and could arise from impurities in phenol used for extraction

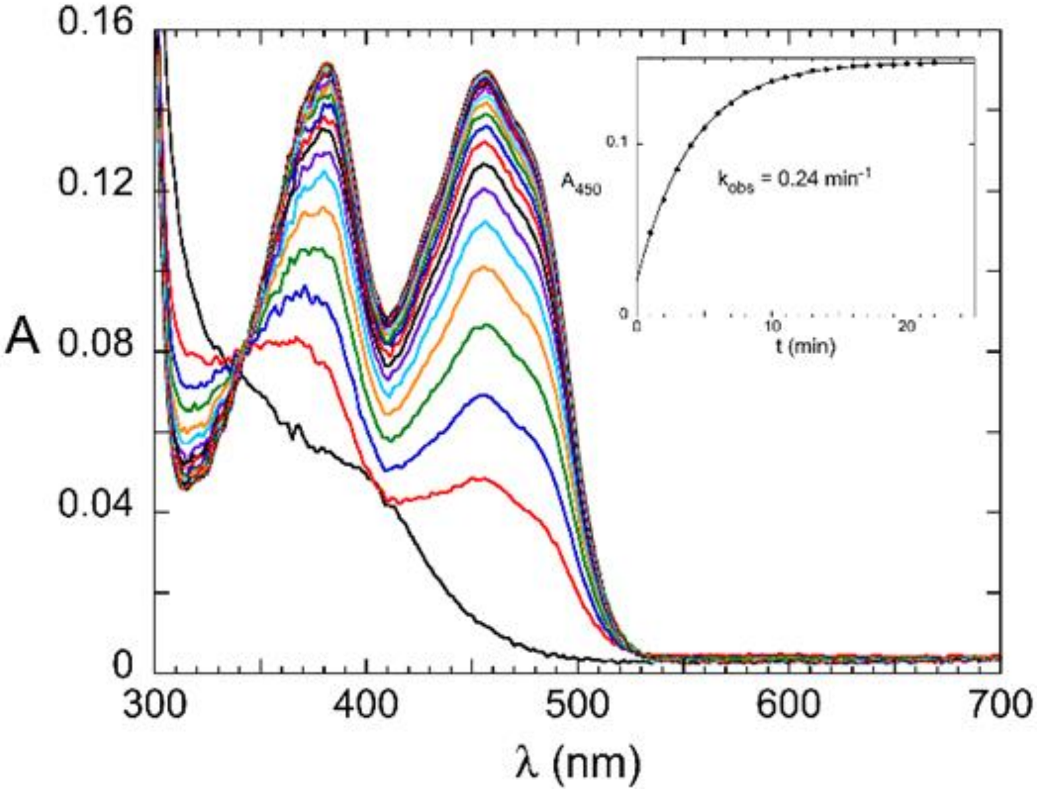

Supplementary Figure 5: Reaction of ThyX with formaldehyde. An anaerobic solution of the thyX•dUMP complex was titrated to complete reduction (2-electrons/FAD) with dithionite. A concentrated solution of formaldehyde was added anaerobically, and UV/visible spectra were scanned every minute in a scanning spectrophotometer. Oxidized enzyme was produced over the course of ~20 minutes at this formaldehyde concentration; its appearance fit a single exponential (inset).

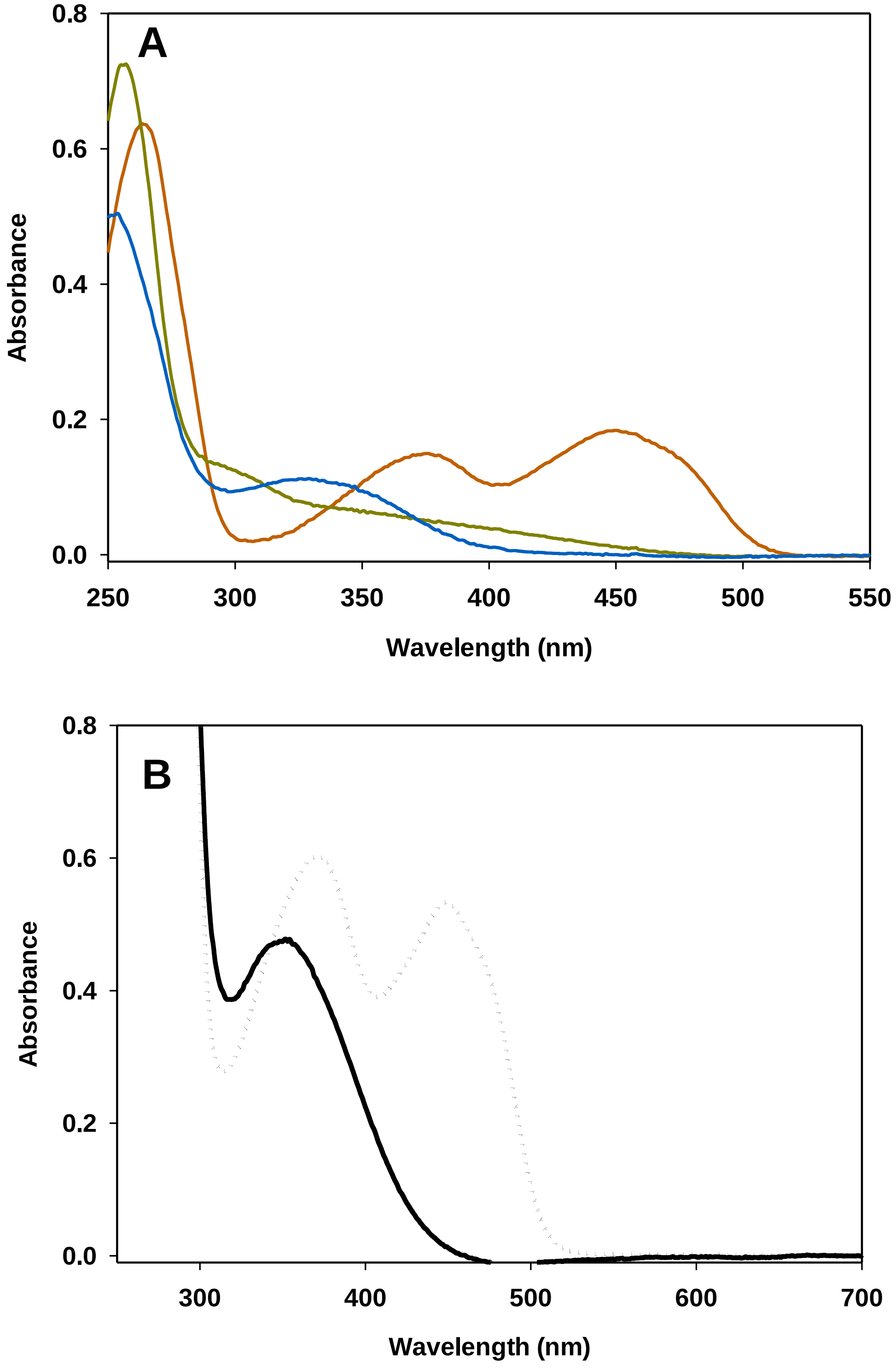

Supplementary Figure 6: Reaction of free reduced FAD with formaldehyde and reconstitution with apoThyX. A) Absorbance spectrum of 15 µM free FAD (orange), reduced FAD with 1 molar equivalent dithionite (green) or incubated with 100 mM CH_2O (blue). B) Absorption of reconstituted ApoThyX with the carbinolamine FAD adduct after PD-10 desalting column in the absence of dUMP (solid line) or in the presence of 1 mM dUMP (dotted line).

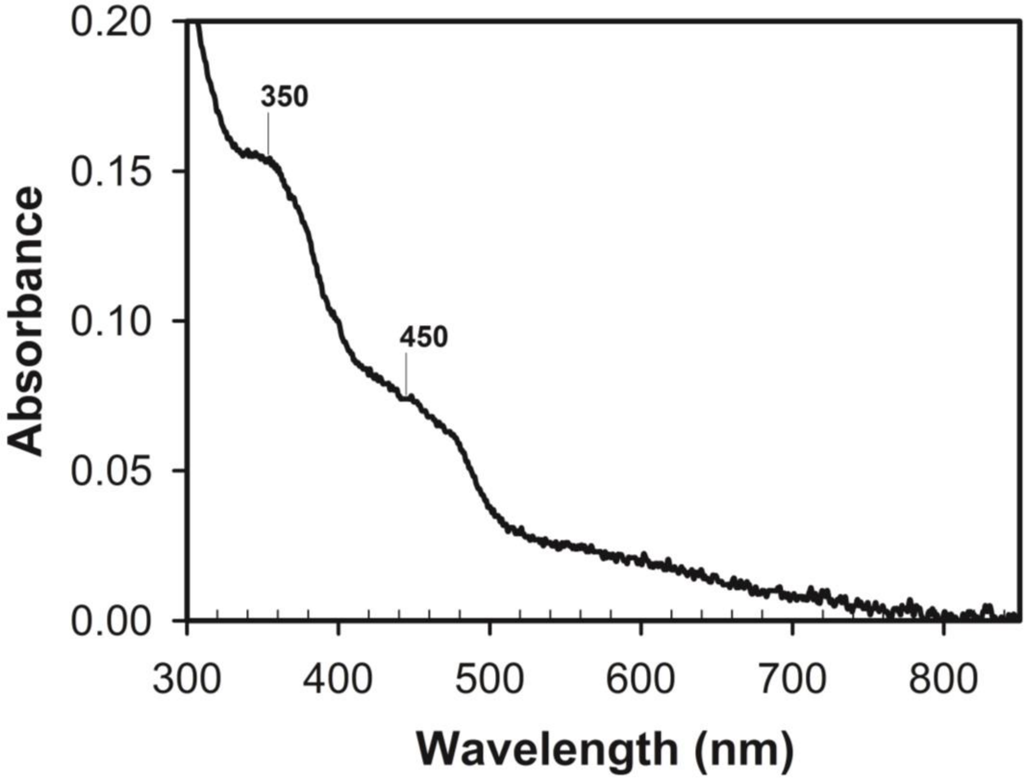

Supplementary Figure 7: Absorbance spectrum of crystal of ThyX in complex with the synthetic carbinolamine FAD derivative.

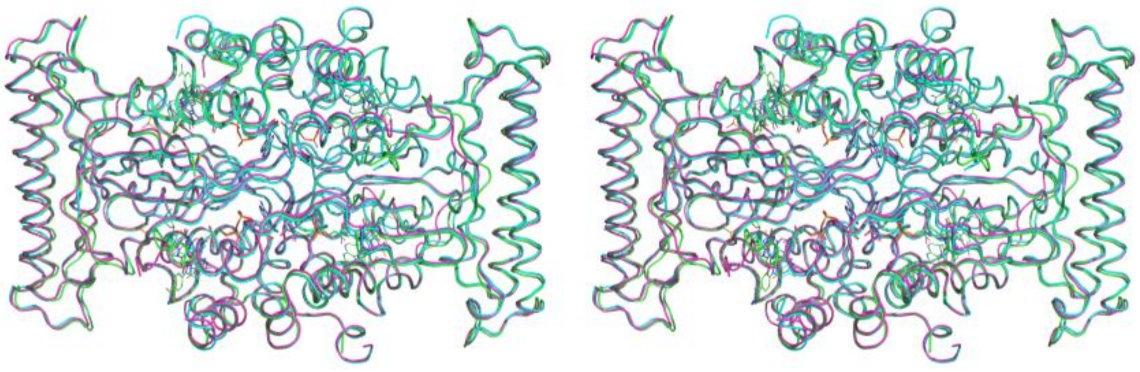

Supplementary Figure 8: Stereo-view of structural comparison of apoThyX/synthetic 4' (violet), $FADH^{-}$•CH_2O (cyan) and ThyX in complex with CH_2THF and dUMP (pdb, 4gt9, green).

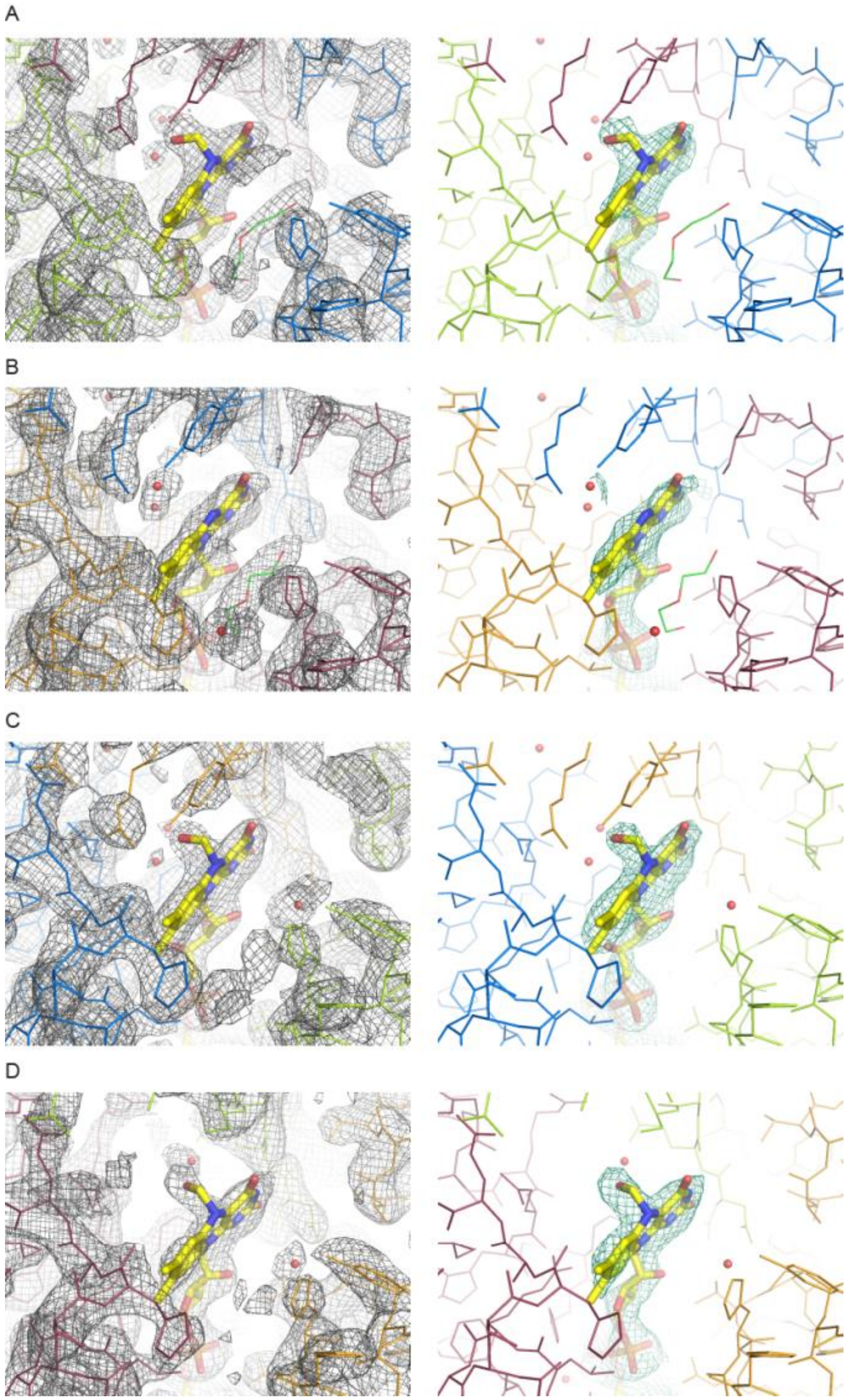

Supplementary Figure 9: Section of the *$2mF_0$-DF_c* electron density (left) and omit map (right) contoured at 1 σ of the four flavin binding sites observed in the asymmetric unit of apoThyX/synthetic 4'. Chains A,B,C,D are colored in limon, orange, blue and brown respectively. Flavin is represented as yellow sticks.

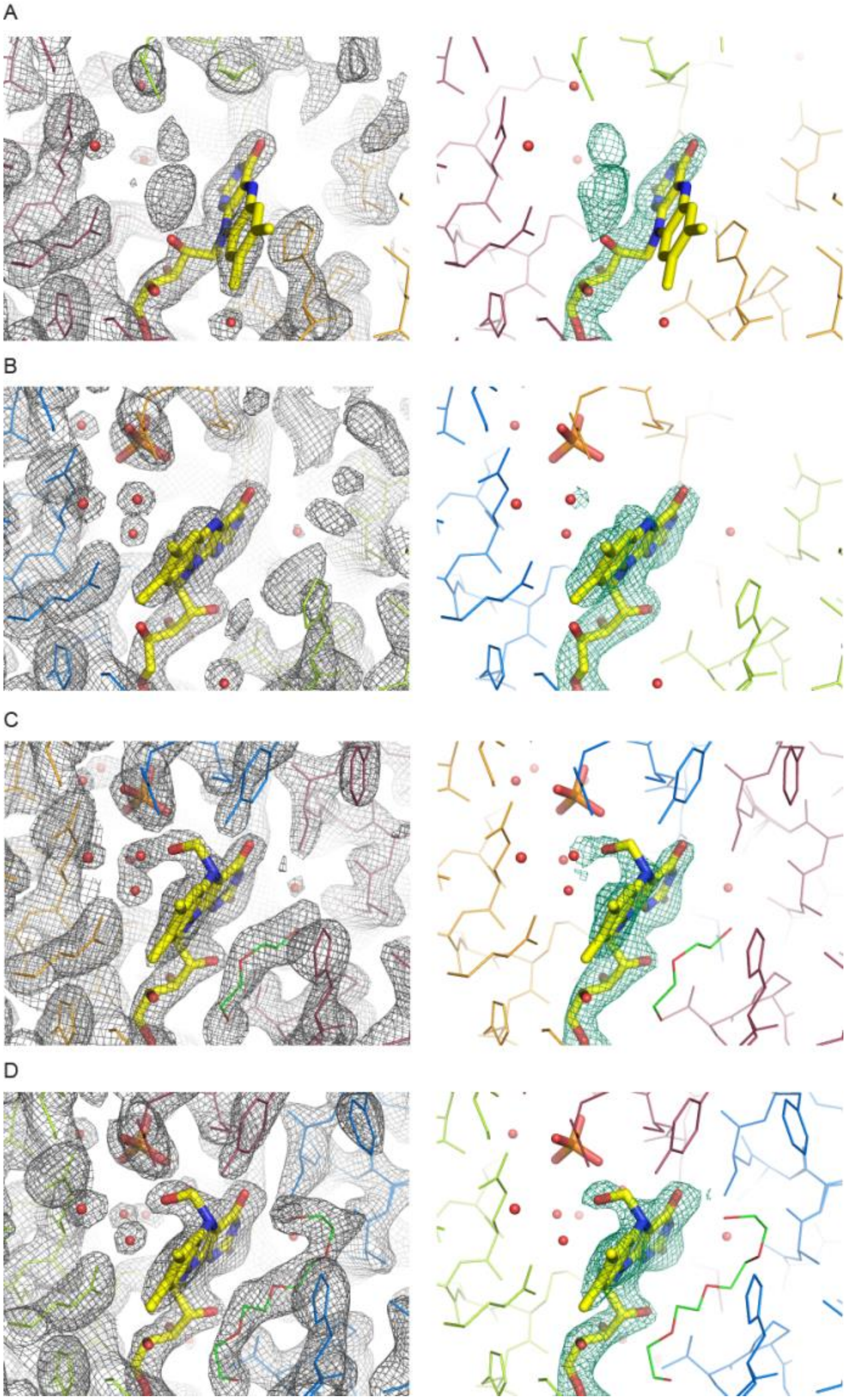

Supplementary Figure 10: Section of the *$2mF_0$-DF_c* electron density (left) and omit map (right) contoured at 1 σ of the four flavin binding sites observed in the asymmetric unit of $FADH^-$ •CH_2O. Chains A,B,C,D are colored in limon, orange, blue and brown respectively. Flavin is represented as yellow sticks. The conformation of the flavin in chain A is different from the one observed in the other three chains. Moreover, the electron density of this flavin is weak suggesting an alternative conformation that could not reliably be modeled.

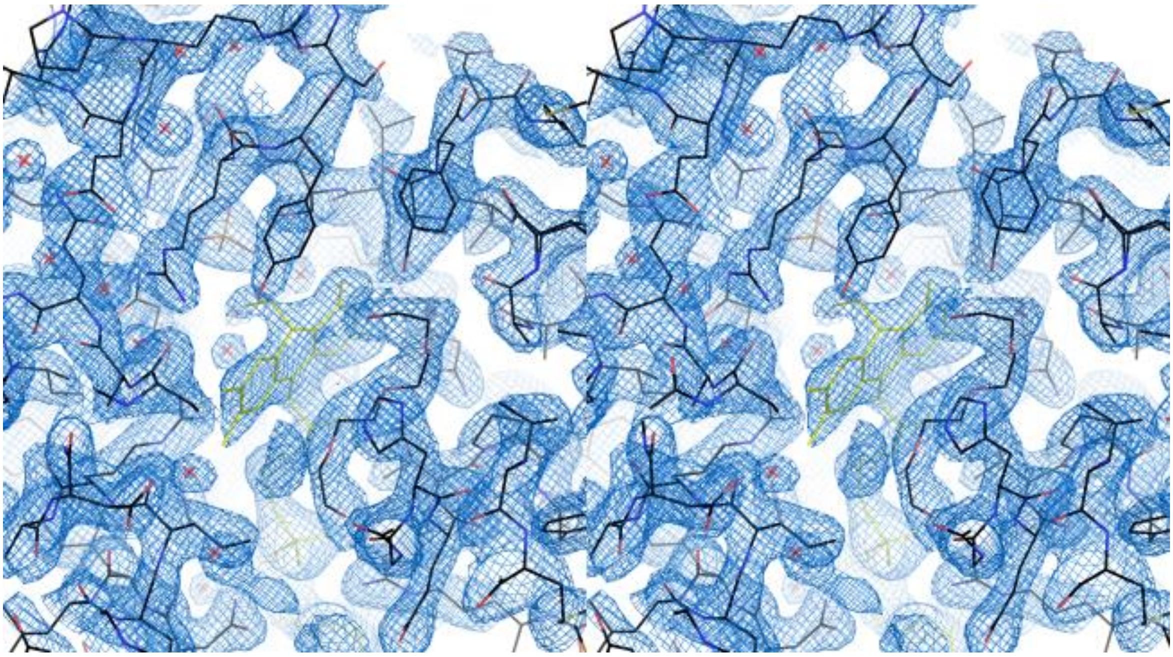

Supplementary Figure 11: Section of the electron density *2mF0-DFc* contoured at the 1σ level around one 4' in the crystal of $FADH^{-}$•CH_2O. The flavin is shown in yellow. For the protein C, N, O atoms are colored black, blue and red respectively.

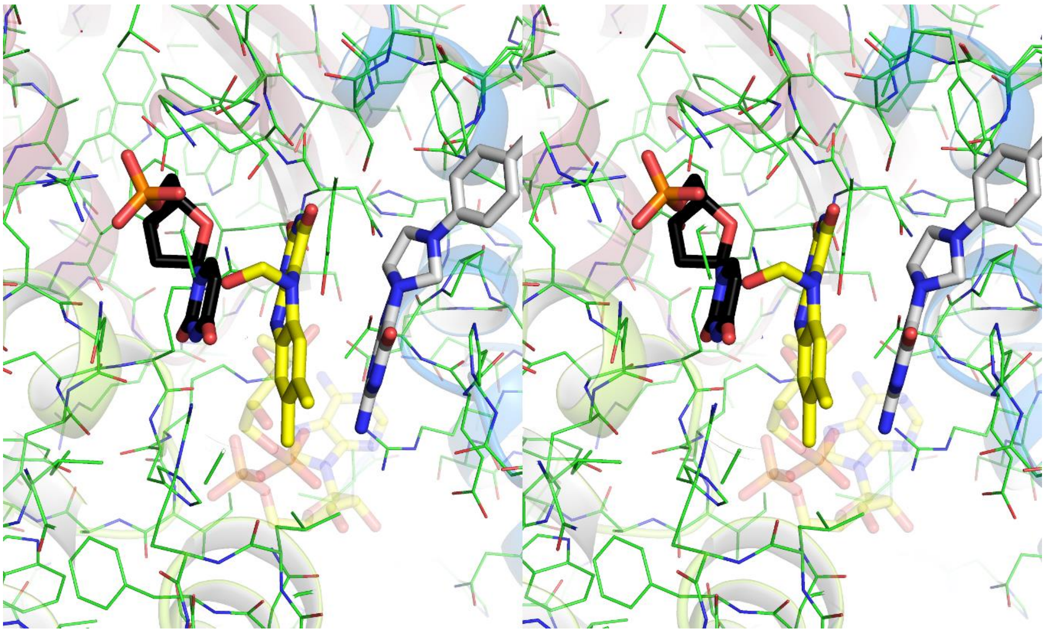

Supplementary Figure 12: Stereo view of a structural model of ThyX in complex with the flavin carbinolamine, dUMP and folate. The model was obtained by superimposing the crystal structure of $FADH^{-}$•CH_2O complex with the structure of ThyX in complex with dUMP and folate (pdb, 4gt9). The structures used for this model are: (i) ThyX-$FADH^{-}$ soaked with 20 mM CH_2O vs (ii) pdb 4gt9

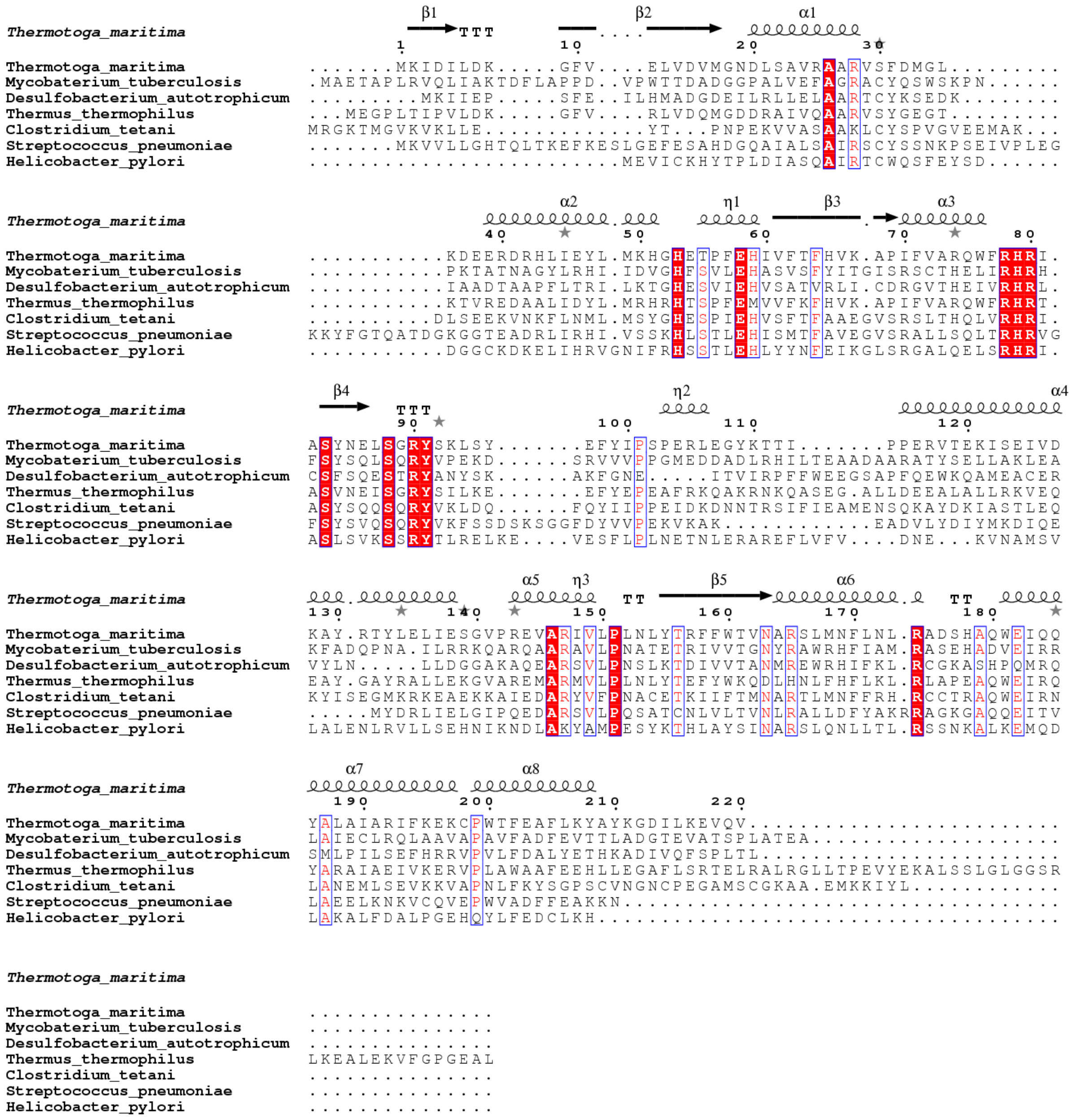

Supplementary Figure 13. Primary sequence alignment of ThyX enzymes. Conserved residues are highlighted in red. The secondary structure of Thermotoga maritima is indicated on top of the sequences. S88 and Y91 proposed to facilitate the elimination of the β-hydroxyl leaving group of the flavin carbinolamine are strictly conserved in ThyX enzymes.

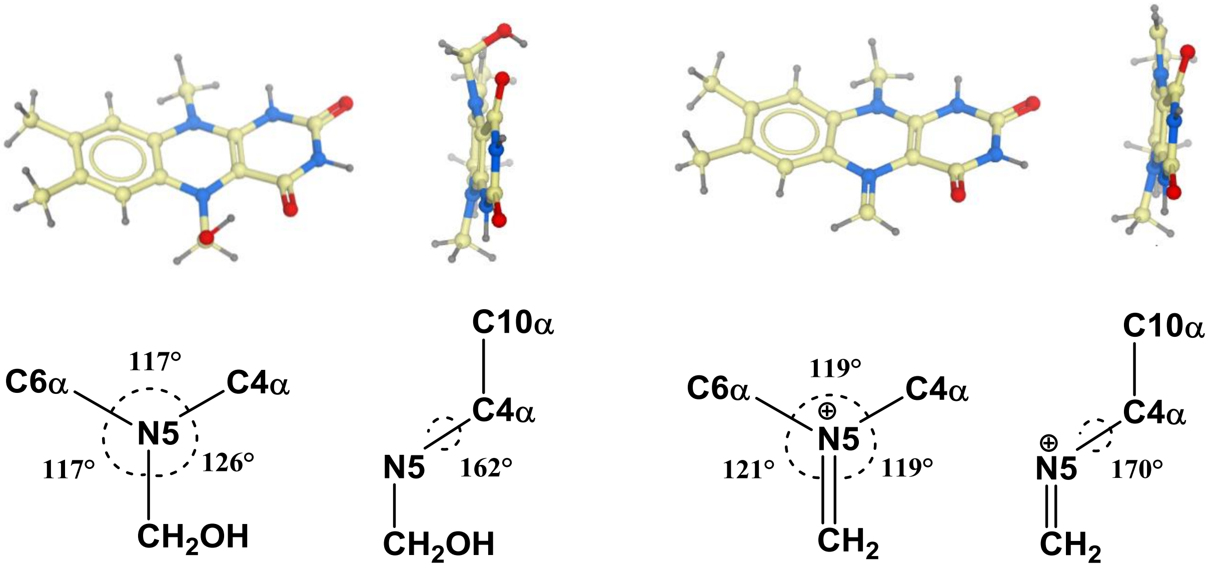

Supplementary Figure 14: Geometric comparison of a N5 carbinolamine and iminium flavin. a and b, 3D-model of N5-carbinolamine and N5-iminium adduct of lumiflavin in two different orientation, respectively. Below each model is shown the planar and torsion angles of each N5-adduct.

Supplementary Table 1: Data collection and refinement statistics (molecular replacement)

	ThyX-FADH$_2$ + soaking with 20 mM Formaldehyde	ThyX + N5(hydroxymethyl)-FAD
PDB	7NDW	7NDZ
**Data collection**		
Space group	$P2_12_12_1$	$P2_12_12_1$
Wavelength (Å)	0.9801	0.9801
Cell dimensions		
a, *b*, *c* (Å)	54.96, 116.78, 141.06	55.08, 117.56, 142.19
α, β, γ (°)	90, 90, 90	90, 90, 90
Resolution (Å)	46.90 - 2.00 (2.07 -2.00)	58.78 – 2.70 (2.80 -2.70)
R_{merge}	0.086 (1.682)	0.162 (1.245)
R_{meas}	0.098 (1.891)	0.183 (1.396)
R_{pim}	0.045 (0.854)	0.083 (0.622)
CC½	0.998 (0.375)	0.994 (0.486)
<I / σI>	13.6 (0.5)	5.5 (0.8)
Wilson B factor	49.76	91.01
Completeness (%)	98.4 (95.4)	99.58 (99.92)
Redundancy	4.59 (4.75)	4.7 (5.0)
**Refinement**		
Resolution (Å)	44.98– 2.00	40.83 – 2.70
No. reflections	61503	26018
R_{work} / R_{free}	0.208 (0.269) / 0.231 (0.327)	0.222 (0.239) / 0.247 (0.242)
No. atoms		
Protein	6896	6854
Ligands	293	242
Water	151	37
B-factors		
Protein	55.82	74.33
Ligand/ion	55.83	63.81
Solvent	58.03	58.70
R.m.s. deviations		
Bond lengths (Å)	0.010	0.008
Bond angles (°)	1.04	0.98

*Data were collected on a single crystal. Values in parentheses are for highest-resolution shell.

Supplementary References

1 Koehn, E. M. *et al.* Folate binding site of flavin-dependent thymidylate synthase. *Proceedings of the National Academy of Sciences of the United States of America* **109**, 15722-15727, doi:10.1073/pnas.1206077109 (2012).

2 Mishanina, T. V. *et al.* An unprecedented mechanism of nucleotide methylation in organisms containing thyX. *Science* **351**, 507-510, doi:10.1126/science.aad0300 (2016).
