# Supplementary material for: An enzymatic activation of formaldehyde for nucleotide methylation
Source: Nat Commun. 2021 Jul 27;12:4542. doi: 10.1038/s41467-021-24756-8 (PMC8316439; doi:10.1038/s41467-021-24756-8)
Supplement: Supplementary file 3 — Reporting summary [file 41467_2021_24756_MOESM3_ESM.pdf]

nature research

Corresponding author(s): Djemel Hamdane

Last updated by author(s): Jun 30, 2021

Reporting Summary

Nature Research wishes to improve the reproducibility of the work that we publish. This form provides structure for consistency and transparency in reporting. For further information on Nature Research policies, see our Editorial Policies and the Editorial Policy Checklist.

Statistics

For all statistical analyses, confirm that the following items are present in the figure legend, table legend, main text, or Methods section.

n/a	Confirmed	
x	☐	The exact sample size (*n*) for each experimental group/condition, given as a discrete number and unit of measurement
x	☐	A statement on whether measurements were taken from distinct samples or whether the same sample was measured repeatedly
x	☐	The statistical test(s) used AND whether they are one- or two-sided<br>*Only common tests should be described solely by name; describe more complex techniques in the Methods section.*
x	☐	A description of all covariates tested
x	☐	A description of any assumptions or corrections, such as tests of normality and adjustment for multiple comparisons
x	☐	A full description of the statistical parameters including central tendency (e.g. means) or other basic estimates (e.g. regression coefficient) AND variation (e.g. standard deviation) or associated estimates of uncertainty (e.g. confidence intervals)
x	☐	For null hypothesis testing, the test statistic (e.g. *F*, *t*, *r*) with confidence intervals, effect sizes, degrees of freedom and *P* value noted<br>*Give P values as exact values whenever suitable.*
x	☐	For Bayesian analysis, information on the choice of priors and Markov chain Monte Carlo settings
x	☐	For hierarchical and complex designs, identification of the appropriate level for tests and full reporting of outcomes
x	☐	Estimates of effect sizes (e.g. Cohen's *d*, Pearson's *r*), indicating how they were calculated

Our web collection on statistics for biologists contains articles on many of the points above.

Software and code

Policy information about availability of computer code

Data collection	In-house data collection software (MxCuBE) was provided by PROXIMA-2 beamline at the SOLEIL synchrotron Saint-Aubin, France. Kinetic Studio 5.10.6; UV Probe 2.70; FlexControl software package (Bruker Daltonics); Scan win UV Carry, varian; In-cristallo UV/Vis microspectrophotometry OOIBase32 software (Ocean Optics); Bruker Avance-III NMR software
Data analysis	The X-ray diffraction data were indexed, integrated and scaled using XDS. Molecular replacement was performed with Phaser and refinement was done using Buster. Model building was performed using Coot. KaleidaGraph 4.5.4; PyMOL 2.3.5; SigmaPlot version 12.0; FlexAnalysis software package (Bruker Daltonics)

For manuscripts utilizing custom algorithms or software that are central to the research but not yet described in published literature, software must be made available to editors and reviewers. We strongly encourage code deposition in a community repository (e.g. GitHub). See the Nature Research guidelines for submitting code & software for further information.

Data

Policy information about availability of data

All manuscripts must include a data availability statement. This statement should provide the following information, where applicable:

- Accession codes, unique identifiers, or web links for publicly available datasets
- A list of figures that have associated raw data
- A description of any restrictions on data availability

NDZAtomic coordinates and structure factor amplitudes for the crystal structures have been deposited at the Protein Data Bank under accession code 7NDZ and 7NDW All other data generated or analyzed during this study are included in this published article and its supplementary information files and are available from the corresponding authors upon reasonable request.

Field-specific reporting

Please select the one below that is the best fit for your research. If you are not sure, read the appropriate sections before making your selection.

☒ Life sciences ☐ Behavioural & social sciences ☐ Ecological, evolutionary & environmental sciences

For a reference copy of the document with all sections, see nature.com/documents/nr-reporting-summary-flat.pdf

Life sciences study design

All studies must disclose on these points even when the disclosure is negative.

Sample size	One crystal sample bound to N5(hydroxymethyl)-FAD and one crystal sample bound to $FADH_2$ and soaked with 20 mM formaldehyde. Sample sizes were chosen based on convention in the field. These sample sizes were sufficient given the robust signal changes measured in the experiments.
Data exclusions	No data were excluded.
Replication	Measurements were repeated at least two times and all were reproducible.
Randomization	Randomization was not required for any of the experiments in this study.
Blinding	Blinding was not required for any of the experiments in this study.

Reporting for specific materials, systems and methods

We require information from authors about some types of materials, experimental systems and methods used in many studies. Here, indicate whether each material, system or method listed is relevant to your study. If you are not sure if a list item applies to your research, read the appropriate section before selecting a response.

Materials & experimental systems

n/a	Involved in the study	
☒	☐	Antibodies
☒	☐	Eukaryotic cell lines
☒	☐	Palaeontology and archaeology
☒	☐	Animals and other organisms
☒	☐	Human research participants
☒	☐	Clinical data
☒	☐	Dual use research of concern

Methods

n/a	Involved in the study	
☒	☐	ChIP-seq
☒	☐	Flow cytometry
☒	☐	MRI-based neuroimaging
